# Solving the Issue of Discriminant Roughness of Heterogeneous Surfaces Using Elements of Artificial Intelligence

**DOI:** 10.3390/ma14102620

**Published:** 2021-05-17

**Authors:** Milena Kubišová, Vladimír Pata, Dagmar Měřínská, Adam Škrobák, Miroslav Marcaník

**Affiliations:** Faculty of Technology, Tomas Bata University in Zlín, Vavrečkova 275, 760 01 Zlín, Czech Republic; pata@utb.cz (V.P.); merinska@utb.cz (D.M.); skrobak@utb.cz (A.Š.); marcanik@utb.cz (M.M.)

**Keywords:** surface quality, metallic materials, statistical analysis of measured data, perceptron

## Abstract

This work deals with investigative methods used for evaluation of the surface quality of selected metallic materials’ cutting plane that was created by CO_2_ and fiber laser machining. The surface quality expressed by Rz and Ra roughness parameters is examined depending on the sample material and the machining technology. The next part deals with the use of neural networks in the evaluation of measured data. In the last part, the measured data were statistically evaluated. Based on the conclusions of this analysis, the possibilities of using neural networks to determine the material of a given sample while knowing the roughness parameters were evaluated. The main goal of the presented paper is to demonstrate a solution capable of finding characteristic roughness values for heterogeneous surfaces. These surfaces are common in scientific as well as technical practice, and measuring their quality is challenging. This difficulty lies mainly in the fact that it is not possible to express their quality by a single statistical parameter. Thus, this paper’s main aim is to demonstrate solutions using the cluster analysis methods and the hidden layer, solving the problem of discriminant and dividing the heterogeneous surface into individual zones that have characteristic parameters.

## 1. Introduction

The surface quality of the solid material determines and expresses both the grade of the workpiece and the product. Wood and stone products already require a certain level of surface quality for further use. Requirements on it are intensified with the development of metallurgy, especially in the production of cutting tools for daily use, e.g., knives, swords, sickles, scythes, and tools. However, in the production of these types of tools, the evaluation of the surface by sight or touch has been sufficient [1].

Machining methods have also been evolving, ranging from downright primitive to the traditional methods used to date, along with methods for classifying surface quality determination [1,2]. The roughness began to be prescribed in the blueprint and was given by standards that became documents approving the rules of product reproducibility. The “measurement” of surface roughness by sight and touch was no longer sufficient because it could not be measured accurately and precisely enough. With the advent of new technologies and modern measuring instruments, it was possible to measure the surface roughness and determine its parameters more accurately [1,2]. These technical possibilities were reflected in the standardization process [2].

The methods of surface quality evaluation prescribed by the standards and the repeatability of the production process have led to the idea that it is possible to determine the type of material to be machined under the conditions of repeatability of the measured roughness parameters. The subject of this work was to verify this idea [3,4].

The need for standardization of parts, products, and processes in industrial production is almost as old as industrial production itself. However, as globalization increases, the importance of interchangeability and substitutability within the supplier–customer chain increases. Setting up rules for assessing different products or production processes is the main goal of the International Organization for Standardization. Standardization in surface quality assessment is mainly dealt with in ISO 4287 and ISO 4288 [2].

Therefore, the article’s aim, considering the previous introduction, is to independently evaluate the surfaces that have arisen as a result of applying a water jet and laser beam to the aforementioned surfaces [5]. Due to the energy loss during the passage, when the cutting is carried out, the quality of the cut surface changes, which can be described by the roughness or waviness parameters, both in 2D and 3D [2].

Therefore, it is clear that this is a classical discriminatory task that methods of discrimination or cluster analysis can solve. However, in this case, the main responsibility for the solution is transferred from the metrologist to the statistician, which may be convenient for scientific but not practical application [6].

Normally, this problem is solved by evaluation of only the Ra parameter. Unfortunately, it has been confirmed several times that the parameter Ra itself is not sufficiently indicative of the surface quality. Therefore, the amplitude parameters Rz and the hybrid parameter Rmr(c) were added to the neural network [7].

The novelty of this article lies in the possibility of comparing the roughness of surfaces created by two different machining methods by using a neural network. The surface quality expressed by Rz and Ra’s roughness parameters is examined depending on the sample material and the machining technology that was used [1,2]. The next part deals with the use of neural networks to evaluate measured data. In the last part, the measured data were statistically evaluated [2,8]. Based on the conclusions of this analysis, the possibilities of using neural networks to determine the material of a given sample with known roughness parameters were evaluated [8].

The suitability of using the perceptron neural networks with one hidden layer for this exact purpose will be described in the article. The suitability of using the perceptron neural networks with one hidden layer for this exact purpose will be described in the article. By creating a suitable file containing learning information for the neural network and finding a suitable number of neurons in a hidden layer, it is possible to apply it to solve the discriminatory task and, more precisely, find individual parameters [9,10].

## 2. Materials and Methods

### 2.1. Studied Materials and Their Machining

Three types of materials were used for the measurements, namely structural steel 1.0038- S235JRC + N (sample 235), abrasion-resistant stainless steel HARDOX 450 (sample HARDOX), and stainless steel 1.0043-X5CrNi18-10 (sample 1_430). 

Information data about the machining methods, machining process, and machining conditions of the samples can be seen in Table 1. Then, Table 2 shows the abbreviations that are used in the article to identify the sample.

### 2.2. Surface Quality 

During machining, surface irregularities arise due to the production technology. The quality of the surface and the associated roughness directly depend on the modifying method. To make surfaces comparable in quality, ISO 4287, ISO 4288, ISO 25178, and other norms define a number of parameters that can be used in other to quantify the surface of an observed sample [11].

This article presents parameters concerning surface roughness, i.e., R parameters, which were measured by Talysurf CLI 500 profilometer (Taylor & Hobson, Leicester, United Kingdom). These parameters include Ra—Arithmetical mean of height and Rz—Maximum height of profile. Furthermore, one hybrid parameter, Rmr (c)—Load length ratio of profile curve elements to the evaluation length at cut level c (% or µm), was used [1,5].

Before the roughness was measured on a Talysurf profilometer, the machined-surface areas were checked on a Leica optical microscope with a magnification of 50×. This control is necessary for the prevention of errors in the measurement itself. ISO 4287 clearly specifies that surface damage cannot be assessed.

**Figure 1:**
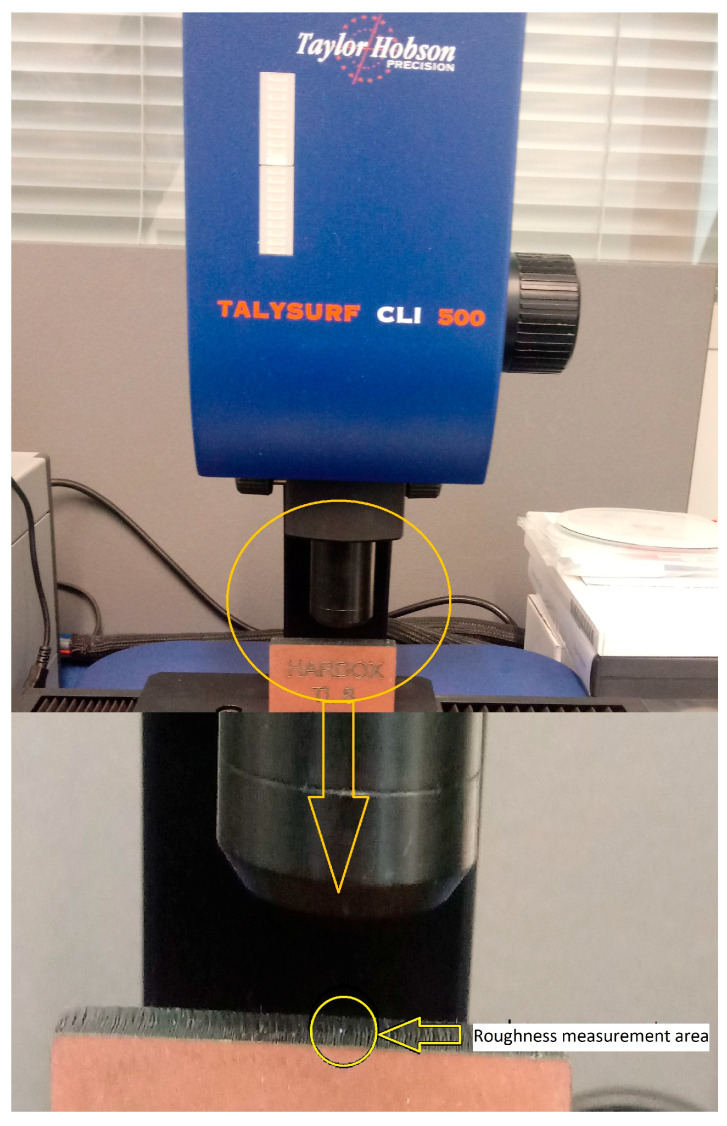
Profilometer Talysurf CLI 500.

The measurement was performed under laboratory measurement conditions (temperature: 22 °C, atmospheric pressure: 1045 hPa) on a Talysurf CLI 500 profilometer (Figure 1). From three materials and two types of machining, six samples were produced. For each sample, 160 cuts were done on the X-axis and 280 on the Y-axis. The disproportion in the performed cuts is given by ISO 4277, where the main emphasis is placed on the X-axis as the central axis.

The following scanning parameters on the Talysurf CLI 500 profilometer were set: intensity reflection in the range of 85–95% (stabilization of the Z-axis), measurement speed 50 µm/s, and measurement spacing 5 µm on X and Y axes. Figure 1 shows the Talysurf profilometer with the marked measuring area.

### 2.3. Statistical Tools for Data Evaluation

On a theoretical level, surface quality is assessed by using a parameter that is given by ISO 4287 and ISO 25178 standards, and conclusions are drawn from statistical tests and analyses. These tests are standard statistical hypothesis tests, based on the assumption that the data from the scanned surface show a normal distribution and can be neglected [8,12]. Furthermore, the effect of systematic errors is not considered. Therefore, data analysis is carried out first to determine the type of distribution, deviations, skewness, and sharpness. The arithmetic mean and variance are mainly evaluated for parameters of the dataset [13,14].

### 2.4. Data Classification

In assessing the measurement data from a plurality of samples, data must be split into several subsets according to specific criteria. This identifies groups that show some similarity [8,12]. The division of data into groups is a part of the “statistical learning” discipline, where, based on a set of so-called training data, a prediction model is established. This allows a prediction of the output value with a certain probability. Further described is the use of neural networks to precisely determine the resulting values based on the input measurement data and cluster analysis method [9,12].

Clustering is one of the multidimensional data analysis techniques nowadays frequently used in artificial intelligence. The basic idea of the method is to divide the measured data into clusters, so the items from one cluster have “more similar” properties than others [1,14]. Furthermore, this approach is based on the evaluation of measured parameters (Ra, Rz, and Rmr) of samples’ surfaces (F_1_430, F_235, HARDOX) that were machined by laser technology CO_2_ and fiber [6,8].

For this distribution, the approach by Ward that is capable of finding the nearest clusters by optimizing distances of individual clusters was used. The optimization is based on finding the correlation coefficients that are linear scale distances [8,15].

The correlation coefficient used in the article expresses the mutual linear relationship between two quantities describing the quality of a surface. Then, the degree of correlation is expressed by the Pearson correlation coefficient, which can take values in the closed interval ranging from −1 to +1. A value close to +1 (−1) means that the linear relationship between two quantities is “very probable”. Using cluster analysis, it is possible to demonstrate that two (or more) clusters are “more probable” than the others [8,15].

### 2.5. Mathematical Model of the Neuron

**Figure 2:**
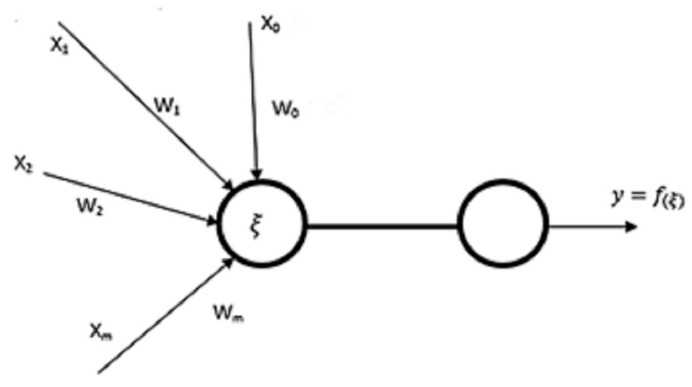
Basic principle of neuron.

In principle, the neuron is a unit that performs the representation of Euclidean space on real numbers {R}n –> R. This means that it converts n inputs to which weights are assigned to one output function value. The neuron output is a value ϒ; which is a function; designated as:

(1) $$\xi =\overset{n}{\underset{i=1}{\sum }}\mathrm{xi}\cdot \mathrm{wi}-\theta$$

where ξ is the intrinsic potential of a neuron. This function is calculated when the sum of the product value input xi × wi exceeds a predetermined value θ called the threshold. Figure 2 shows the principle of a neuron [3,4,13].

The transfer function can be divided into two categories. It is either a leap, i.e., the value of the output changes abruptly when the threshold is exceeded, or analogue, i.e., the continuous transfer function generates the output value. The jump function is only used for the output layer when binary output is required. For other layers, analogy functions are used [4,11].

One neuron itself can perform only the most basic operations. On the contrary, a structure of neurons can perform complex decision-making operations. These structures are known as neural networks. Thus, a neural network is an oriented graph with input, output, hidden nodes, and rated edges representing the signal flow [1,2].

Separate neurons are located in individual nodes. Nodes, their deployment, and interconnections are critical aspects of the network architecture. Input nodes mostly only distribute incoming data to all the first hidden layer’s neurons. Furthermore, the network may consist of several hidden layers designed to perform the desired operations as a whole. The output layer acts mainly as a network output interface [5,6,13].

## 3. Results and Discussion

### 3.1. Materials

**Figure 3:**
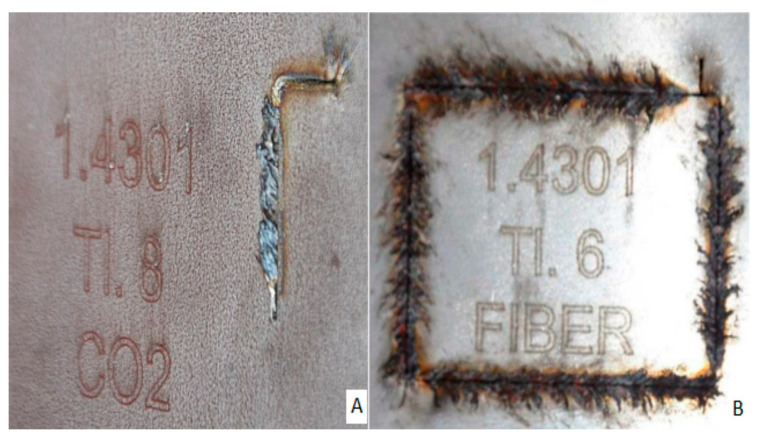
Material cut 1.4301 (**A**), CO_2_ laser, (**B**) Fiber laser.

The material was machined on a CO_2_ laser and a Fiber laser. Figure 3 shows an example of machining samples of material 1.4301 by machining (A) with a CO_2_ laser, and Figure 3B shows the preparation of samples with a fiber laser.

### 3.2. Evaluation of Surface Roughness

As described above, the samples were cut by two types of lasers. First, it was necessary to check the surface on a microscope for defects that prevented a correct roughness assessment.

**Figure 4:**
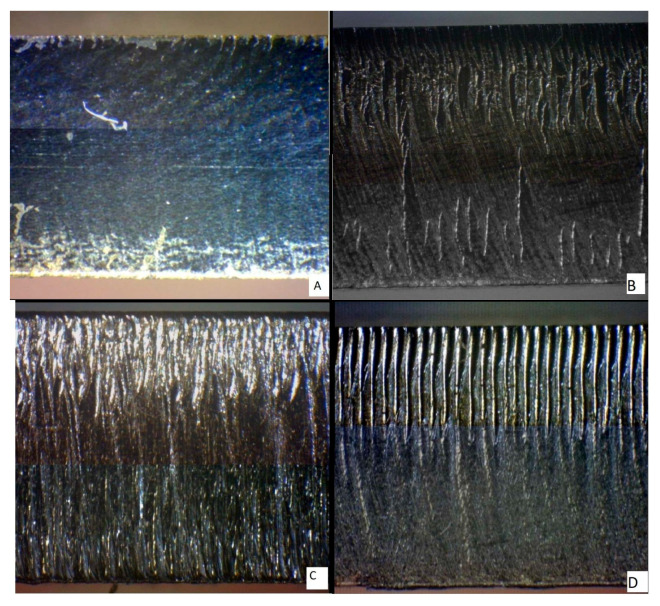
Material cuts 1.0038: (**A**) 6 mm fiber laser, (**B**) 8 mm fiber laser, (**C**) 6 mm CO_2_ laser, (**D**) 8 mm CO_2_ laser.

Figure 4 shows a section of material 1.0038 (6 mm and 8 mm thickness) machined by the fiber laser and CO_2_ laser. As is evident from the figure, the CO_2_ machining left a more apparent mark, while the surface of the sample cut with the fiber laser was smoother.

**Figure 5:**
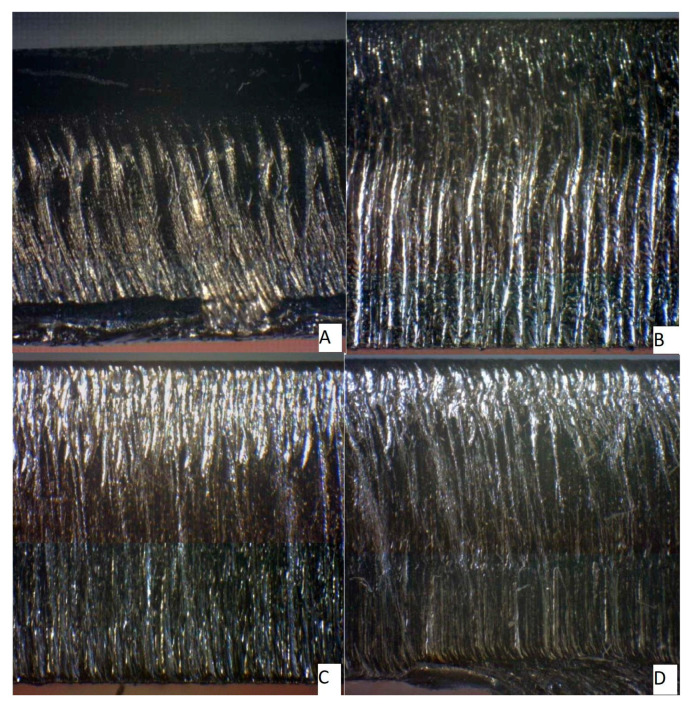
Material cuts 1.4301: (**A**) 6 mm fiber laser, (**B**) 8 mm fiber laser, (**C**) 6 mm CO_2_ laser, (**D**) 8 mm CO_2_ laser.

Figure 5 shows a section of material 1.4301 (6 mm and 8 mm thickness) machined by the fiber laser and CO_2_ laser. The general roughness of sections displayed on this figure is higher than on the previous one. Nevertheless, the surface left by the fiber laser is still smoother than the surface left by the CO_2_ laser. In addition, Figure 5D shows the evidence of the material being melted during the cutting procedure, which could be caused by the higher concentration of nickel in this material.

**Figure 6:**
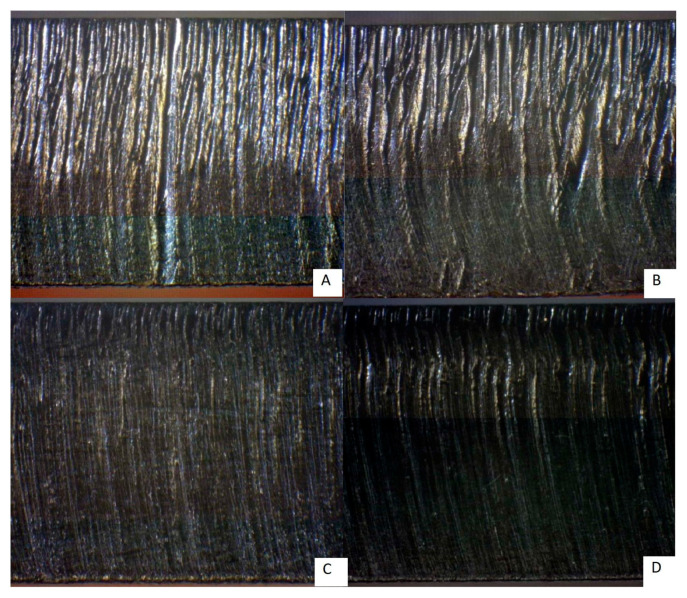
Material cuts Hardox 450: (**A**) 6 mm fiber laser; (**B**) 8 mm fiber laser, (**C**) 6 mm CO_2_ laser; (**D**) 8 mm CO_2_ laser.

Figure 6 shows a section of material Hardox 450 (6 mm and 8 mm thickness) machined by the fiber laser and CO_2_ laser. While the previous two materials had a smoother section surface after being cut by a fiber laser, an opposite trend was observed in samples prepared from HARDOX 450. This might have been caused by the high abrasion resistance of this material.

**Figure 7:**
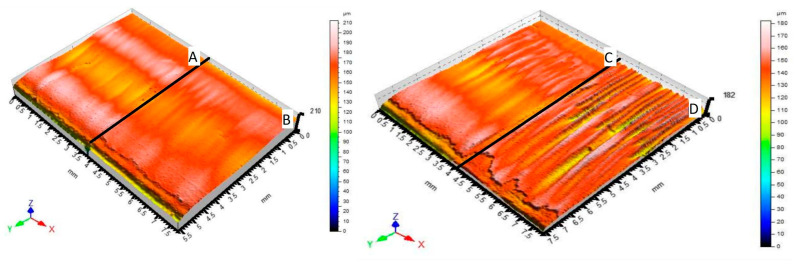
Comparison of 3D scenes of materials 1.0038 section surfaces profilometer.

Figure 7 graphically compares the surfaces of material 1.0038. Figure 7A shows a section of material (6 mm thickness) machined by the fiber laser. Figure 7B shows a section of material (8 mm thickness) machined by the fiber laser, too. Figure 7C shows a section of material (6 mm thickness) machined by the CO_2_ laser. Figure 7D shows a section of material (8 mm thickness) machined by the CO_2_ laser, too. All surfaces are characterized by triple parameters (Ra, Rz, Rmr) that change with the varying measuring position. Examples of measured triplets can be found in Table 5.

**Figure 8:**
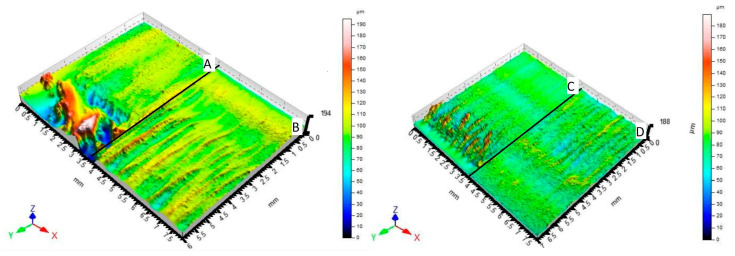
Comparison of 3D scenes of materials 1.4301 section surfaces profilometer.

Figure 8 graphically compares the surfaces of material 1.4301. Figure 8A shows a section of material (6 mm thickness) machined by the fiber laser. Figure 8B shows a section of material (8 mm thickness) machined by the fiber laser. Figure 8C shows a section of material (6 mm thickness) machined by the CO_2_ laser. Figure 8D shows a section of material (8 mm thickness) machined by the CO_2_ laser. All surfaces are characterized by triple parameters (Ra, Rz, Rmr) that change with the varying measuring position. Examples of measured triplets can be found in Table 5.

**Figure 9:**
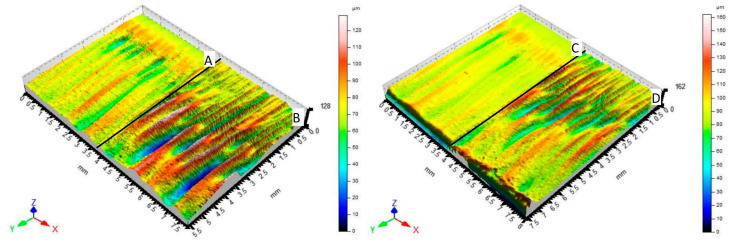
Comparison of 3D scenes of materials Hardox 450 section surfaces profilometer.

Figure 9 graphically compares the surfaces of material Hardox 450. Figure 9A shows a section of material (6 mm thickness) machined by the fiber laser. Figure 9B shows a section of material (8 mm thickness) machined by the fiber laser. Figure 9C shows a section of material (6 mm thickness) machined by the CO_2_ laser. Figure 9D shows a section of material (8 mm thickness) machined by the CO_2_ laser. All surfaces are characterized by triple parameters (Ra, Rz, Rmr) that change with the varying measuring position. Examples of measured triplets can be found in Table 5.

As shown by the figures presented above, the quality of the surfaces of individual materials machined by individual types of lasers is highly variable. In practice, this variability is called a heterogeneous surface. These surfaces are characterized by the fact that the above parameters Ra, Rz, and Rmr show considerable variance. Therefore, it is quite difficult to use classical statistical methods for their recognition and subsequent classification. Therefore, a cluster analysis capturing the similarity of the above-mentioned heterogeneous surfaces seems to be a suitable method. 

### 3.3. Statistical Evaluation

The first step of the statistical evaluation of the measured parameters Ra and Rz of the surfaces of materials 1.0038, 1.4301, and Hardox 450, machined with fiber laser and CO_2_ laser technology, was to perform the Anderson Darling test of normality at the confidence level of 95%. A two-way Grubs test followed this to rule out the possibility of data infiltration by gross errors.

**Figure 10:**
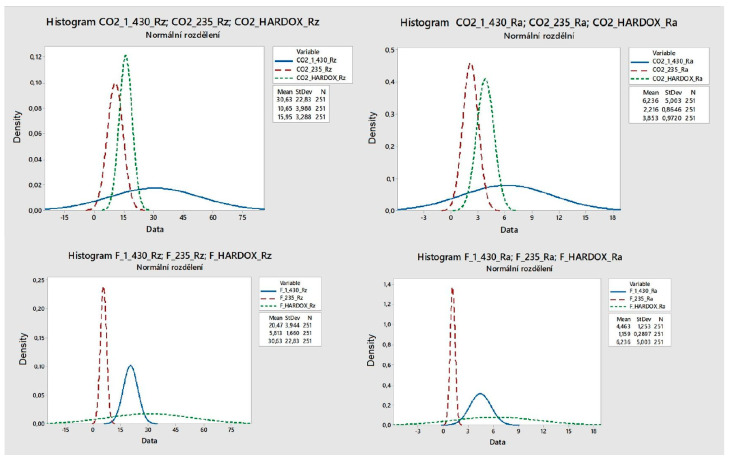
Demonstration of dispersion measuring parameters.

Figure 10 shows the graphs clearly showing that Ra and Rz variances depend on the used machining technology and the material itself.

**Figure 11:**
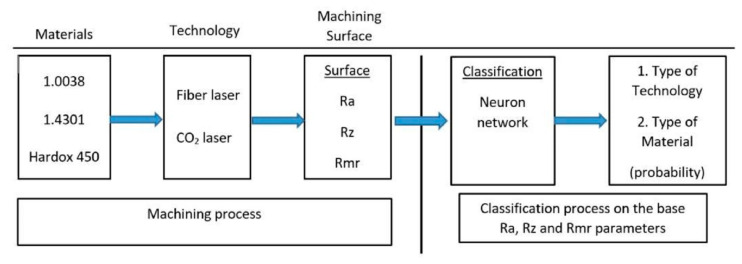
Procedure of classification process using neural network.

For this reason, cluster analysis was applied to demonstrate the similarity of materials and technologies. However, it seems to be much more appropriate for the classification process to use a neural network, which will perform its classification separately after proper “learning” (see Figure 11).

### 3.4. Classification of Evaluated Data and Cluster Analysis

This data analysis step was designed to determine if the data showed any similarity and, if so, to describe this similarity in detail [15]. Since the possibility of determining the material used by the measured roughness parameters was examined, only samples processed with the same technology were compared. For the actual analysis, the recommended Ward’s method was used for the formation of clusters [16].

**Figure 12:**
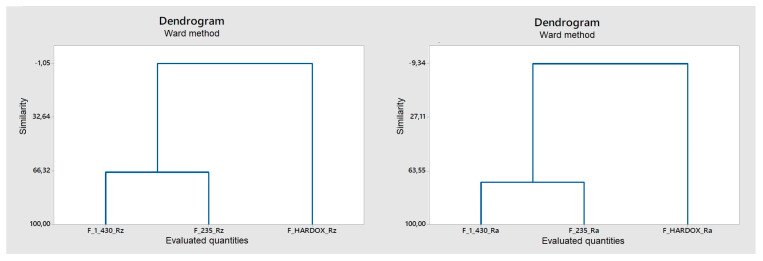
Cluster analysis of samples processed by fiber laser parameters Rz and Ra.

**Figure 13:**
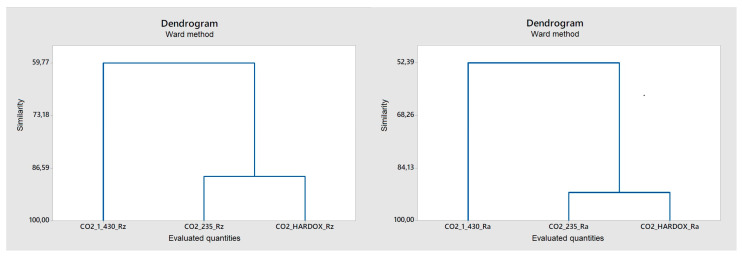
Cluster analysis of samples processed by CO_2_ laser parameters Rz and Ra.

As shown by the dendrograms in Figure 12 and Figure 13, three different sheets of steel, namely 1.0043, 1.0038, and Hardox 450, having a heterogeneous surface resulting from their cutting by two types of lasers, namely a fiber and CO_2_ laser, were considered [17]. The purpose was to find a mutual similarity in terms of discriminant surfaces using the amplitude parameters Ra and Rz. Then, this similarity was numerically expressed using the Pearson correlation coefficient; see Table 3 and Table 4. As expected, F Hardox steel exhibited maximum dissimilarity across clusters [18].

However, the same task is, as it is demonstrated below, was solved more accurately using a single hidden layer neural network where there is no limitation of the linearity given by the Pearson correlation coefficient [19]. Moreover, the amplitude parameters cannot capture the heterogeneous surface property; therefore, it is necessary to add another hybrid parameter (in the sense of ISO 4287), namely Rmr(c), to the inputs of the searched and subsequently tune the neural network [1,6]. 

**Figure 14:**
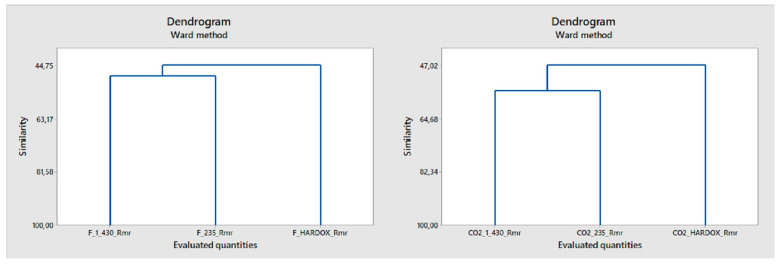
Cluster analysis of samples processed by fiber laser and CO_2_ laser parameter Rmr.

In Table 3 and Table 4, the levels of similarity of fiber and CO_2_ laser-treated samples using Pearson’s correlation coefficient are shown:

This method does not recognize the difference in the cluster of steels 1.0043 and 1.0038 well, so it is necessary to add another discriminatory parameter, the hybrid parameter Rmr (Figure 14).

### 3.5. Neuron Network

Tests have shown that the two materials always have roughness parameters similar to those for which it is not possible to determine a material with sufficient reliability only based on the measured Rz and Ra values. Therefore, the next decision was to consider determining parameter Rmr according to the ISO 4288 norm. For the determination of similarity, we designed a neural network using an adaptation algorithm with one hidden layer.

Software Statistica 14.0 was used to build the neural network. First, the number of neurons in the hidden layer was estimated according to the formula: [1,2]

(2) NNH=(n1+n2)

where:

*NNH* is the number of neurons in the hidden layer
*n*1 is the number of neurons in the input layer
*n*2 is the number of neurons in the output layer

Equation determines the number of neurons in the hidden layer by default [1,2].

To prove the correct functionality of the learned neural network, 30 samples machined with the described technologies were selected. For these samples, the three parameters Rz, Ra, and Rmr were measured in any position. These parameters are entered at the input of the demonstrated neural network, which performed its classification process and calculated the probability of this classification.

From Table 5, it can be concluded that the probability of most classifications exceeds 90% for materials machined with fiber laser. In the case of materials machined with a CO_2_ laser, the triple scattering values result in a lower value of the probability of classification; however, in most cases, it exceeds 60%, which can be considered satisfactory. Should this probability need to be increased, further learning of the neural network would need to be continued.

For each type of machining technology used, a neural network was constructed and tested on a training dataset. This set contained 753 ordered triplets [Rz; Ra; Rmr], which were the measured values of these roughness parameters on all samples machined in the same way. Then, these triplets were inserted at the input of the appropriate neural network, which performed its recognition of the type of material and laser machining technology. The neural network was debugged in the environment of Statistica 14.0 software, specifically in the neural network part.

**Figure 15:**
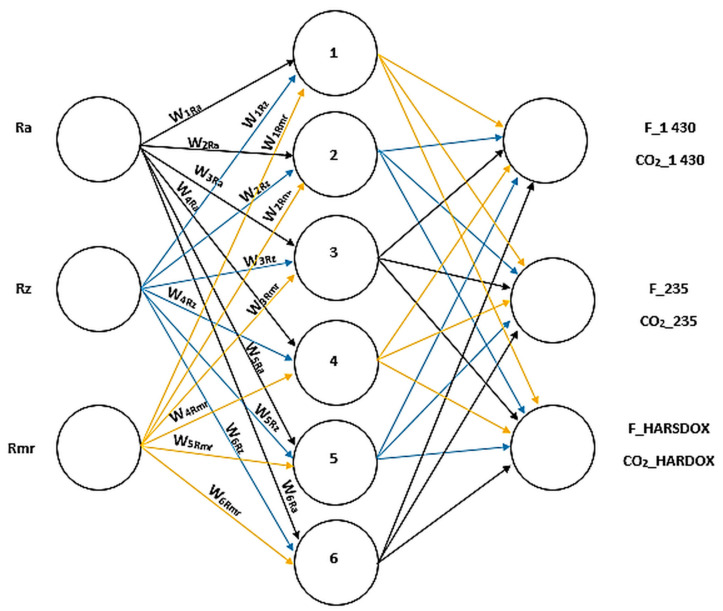
Perceptron with six hidden neurons.

In order to use this neural network without software Statistica 14.0, C ++ source code was generated using the scales, and the neural networks were further tested with a free Dev C ++ development environment. Based on this theoretical estimate, the neural network would look as suggested in Figure 15.

Both neural networks recognized the material correctly, even though the CO_2_ laser sample evaluation neural network showed a lower probability of a correct decision due to a lower level of learning. Hence, a higher error formula value was lying on the error plane.

Based on measurements of individual heterogeneous surfaces of sample materials defined in Table 5 machined with two types of lasers, a database containing 753 arranged triplets, specifically a pair of amplitude parameters Ra and Rz and one hybrid Rmr parameter, was manually created.

This was used to teach the creation of a three-input neural network and with one hidden layer. The optimum number has been optimized by the number 6, which comes from Figure 15 and constitutes the best solution for discrimination, and it is also the network’s output. The result of such discriminant is the specification of the cutting technology on the sample material in any scanned cut of the heterogeneous surface.

As shown in Table 3 and Table 4, the probability of distinguishing the cutting technology from the sample material and the sensing position is rarely below the probability of 85% recognition for the fiber laser and all types of unknown sample materials.

In the case of a CO_2_ laser, the situation is more complicated. The probability of recognition may decrease to 50% for measured unknown samples, which is extreme. In general, samples cut by fiber laser technology have a much higher probability of correct recognition than samples cut by CO_2_ laser, which is mainly due to the different typology of cutting [9,10].

## 4. Conclusions

The main idea of this work was to show the use of the measured values of roughness parameters to determine the sample material using artificial intelligence elements. First, Rz, Ra, and Rmr parameter values were acquired for each sample using the Talysurf CLI500 3D scanner. The measured data were first analyzed by Minitab version 18. It was verified whether the data contained outliers or whether gross measurement errors influenced them in several steps. After that, the sets of values of Rz and Ra’s parameters were analyzed for a statistically significant influence on the material or technology used. In all cases, the influence of the material and the machining technology applied was found to be statistically significant. In the next step, data were sorted by similarity criteria using cluster analysis. Since the two sets of data always shown high similarity, it turned out that using only the parameters Rz and Ra in combination with standard statistical analysis procedures was not sufficient to determine the sample material. Therefore, for each laser type, Statistica 14.0 generated a perceptron neural network with one hidden layer, which evaluated the measured values of the parameters as mentioned above together with the values of the parameter Rmr, which was used as an additional criterion in the determination of the sample. Then, these networks were tested on a test dataset, and in all cases, the material was correctly recognized by the network. 

In general, the fiber laser sample network showed higher reliability than the CO_2_ laser sample network. The highest reliability was recorded for fiber laser-treated steel 1.0043 and the lowest was recorded for CO_2_ laser-treated steel 1.0043.

When comparing the results contained in this article, it can be stated that the evaluation of heterogeneous surfaces of samples based on the parameters of ISO 4287 and ISO 25178 standards cannot be done well without using discriminatory statistical methods. 

However, even today’s commonly used cluster analysis in this assessment may fail or give distorted and inexact results if clustering is based on Pearson coefficients, as described in the article and the relevant tables.

Unambiguously, the best but not the most uncomplicated method seems to be the use of artificial intelligence elements, namely the classical three-input neural network, with one hidden layer containing six neurons, performing its solution to the discriminatory problem in cutting technology and sample material having a heterogeneous surface.

In the case of high-quality sorted data containing triples of amplitude and hybrid parameters, the so-called learning of the found neural network, as described in the article, can be realized according to the triplets describing the character of heterogeneous surfaces in arbitrary sections. As shown from the results table, a well-learned neural network, one with minimized error function, can indicate a low probability of discriminant in various cutting technologies and complicated heterogeneous surfaces.

Then, it depends on whether it is possible to create separate networks for individual technologies, which is understandably more likely to be discriminatory, but at the expense of significantly complicating the solution or a universal network, as demonstrated in this article.

## Figures and Tables

**Table 1:** The laser settings during sample production.

Machining Type	Material Type	Material Thickness [mm]	Cutting Gas	Gas Pres. [Bar]	Cutting Jet Diam. [mm]	Focal Length [mm]	Cutting Speed [mm min^−1^]	Power [W]
Fiber laser	1.0038	6	Oxygen	0.73	1.2	4.4	2600	3080
Fiber laser	1.0038	8	Oxygen	0.5	1.2	4	1700	3050
Fiber laser	Hardox	6	Oxygen	0.73	1.2	4.4	1300	3080
Fiber laser	Hardox	8	Oxygen	0.5	1.2	4	900	3050
Fiber laser	1.4301	6	Nitrogen	17	2.5	−5.7	2100	3080
Fiber laser	1.4301	8	Nitrogen	19.3	2.5	−7.3	1200	3080
CO_2_ laser	1.0038	6	Oxygen	0.7	1	0.7	2700	4000
CO_2_ laser	1.0038	8	Oxygen	0.4	1.5	1.5	2100	4000
CO_2_ laser	Hardox	6	Oxygen	0.7	1	0.7	2700	4000
CO_2_ laser	Hardox	8	Oxygen	0.4	1.5	1.5	2100	4000
CO_2_ laser	1.4301	6	Nitrogen	13	2	−6.5	1350	4000
CO_2_ laser	1.4301	8	Nitrogen	14	2.5	−9	1000	4000

**Table 2:** Marking of measured samples.

Indication	Pattern
F_1_430	steel 1.0043 Fiber laser machining
CO_2__1_430	laser-treated CO_2_ steel 1.0043
F_235	steel 1.0038 Fiber laser machining
CO_2__235	laser-treated CO_2_ steel 1.0038
F_HARDOX	steel HARDOX 450 Fiber laser machining
CO_2__HARDOX	laser-treated CO_2_ steel HARDOX 450

**Table 3:** Similarity levels of fiber laser-treated samples.

Cluster	F_1_430	F_235 F_HARDOX
	Similarity Levels [%]	Similarity Levels [%]
**Rz**	67.3	−1.05
**Ra**	71.5	−9.3

**Table 4:** Similarity levels of laser-treated samples CO_2_.

Cluster	CO_2__1_430	CO_2__235 CO_2__HARDOX
	Similarity Levels [%]	Similarity Levels [%]
**Rz**	59.8	88.7
**Ra**	52.4	91.6

**Table 5:** Proof of the functionality of the proposed neural network solving the classification problem.

Fiber Laser					CO_2_ Laser				
Rz [µm]	Ra [µm]	Rmr	Recognized	%	Rz [µm]	Ra [µm]	Rmr	Recognized	%
20.30	4.80	0.60	F_1_430	0.99850	13.40	2.60	0.62	CO_2__1_430	0.6122
18.50	4.60	1.80	F_1_430	0.99980	11.30	2.50	1.80	CO_2__1_430	0.8978
20.32	4.70	1.20	F_1_430	0.99850	11.90	2,52	1,86	CO_2__1_430	0,7229
20,98	4,78	2,40	F_1_430	0,99820	11,70	2,54	1,24	CO_2__1_430	0,5873
19,40	4,48	3,10	F_1_430	0,99820	12,40	2,40	1,20	CO_2__1_430	0.8348
20.40	4.90	1.80	F_1_430	0.99900	11.60	2.10	0.60	CO_2__1_430	0.8873
19.20	5.00	2.40	F_1_430	0.99930	10.70	2.12	0.62	CO_2__1_430	0.6611
21.80	5.20	1.80	F_1_430	0.99910	11.40	2.62	1.80	CO_2__1_430	0.4669
22.27	5.60	0.60	F_1_430	0.99940	11.20	2.43	0.62	CO_2__1_430	0.5175
21.20	5.20	1.20	F_1_430	0.99920	11.60	2.30	0.62	CO_2__1_430	0.7579
7.50	1.40	3.70	F_235	0.99998	8.70	1.40	1.80	CO_2__235	0.4242
6.80	1.30	3.70	F_235	0.99998	5.00	1.20	13.60	CO_2__235	0.9849
6.70	1.26	1.20	F_235	0.99940	7.30	1.44	4.30	CO_2__235	0.9134
6.60	1.41	1.24	F_235	0.99270	6.20	1.10	3.70	CO_2__235	0.9307
6.20	1.25	0.60	F_235	0.99880	7.50	1.40	3.10	CO_2__235	0.8222
7.20	1.31	1.86	F_235	0.99970	6.70	1.30	3.00	CO_2__235	0.9085
5.90	1.20	8.07	F_235	1.00000	6.00	1.10	3.70	CO_2__235	0.9419
6.00	1.42	3.10	F_235	0.95950	6.60	1.20	0.60	CO_2__235	0.9165
7.20	1.49	1.20	F_235	0.98950	7.10	1.18	2.50	CO_2__235	0.8538
6.95	1.41	0.60	F_235	0.99640	6.30	1.22	8.00	CO_2__235	0.9607
13.30	2.60	0.62	F_HARDOX	0.88750	16.60	3.70	1.80	CO_2__HARDOX	0.6329
11.20	2.50	1.86	F_HARDOX	0.99999	14.10	3.50	1.20	CO_2__HARDOX	0.9273
11.90	2.52	1.80	F_HARDOX	0.99940	15.10	3.60	1.80	CO_2__HARDOX	0.9074
11.80	2.54	1.24	F_HARDOX	0.99900	15.00	3.70	0.62	CO_2__HARDOX	0.9745
12.40	2.41	1.24	F_HARDOX	0.86100	14.44	3.60	1.10	CO_2__HARDOX	0.9728
11.40	2.10	0.62	F_HARDOX	0.87560	14.80	3.50	1.80	CO_2__HARDOX	0.7229
10.80	2.12	0.62	F_HARDOX	0.99880	14.60	3.55	1.24	CO_2__HARDOX	0.8973
11.50	2.62	1.80	F_HARDOX	0.99970	14.20	3.70	0.60	CO_2__HARDOX	0.9937
11.29	2.40	0.60	F_HARDOX	0.99880	15.00	3.50	4.30	CO_2__HARDOX	0.8853
11.64	2.30	0.62	F_HARDOX	0.99870	13.30	3.40	3.70	CO_2__HARDOX	0.9415

## Data Availability

The data presented in this study are available on request from the corresponding author.
